# Is bovine density and ownership associated with human tuberculosis in India?

**DOI:** 10.1371/journal.pone.0283357

**Published:** 2023-03-22

**Authors:** Katriina Willgert, Susie da Silva, Ruoran Li, Premanshu Dandapat, Maroudam Veerasami, Hindol Maity, Mohan Papanna, Sreenidhi Srinivasan, James L. N. Wood, Vivek Kapur, Andrew J. K. Conlan

**Affiliations:** 1 Disease Dynamics Unit (DDU), Department of Veterinary Medicine, University of Cambridge, Cambridge, United Kingdom; 2 Department of Epidemiology, Harvard T.H. Chan School of Public Health, Boston, MA, United States of America; 3 ICAR-Indian Veterinary Research Institute, Eastern Regional Station, Kolkata, West Bengal, India; 4 CisGen Biotech Discoveries Pvt Ltd, Chennai, India; 5 Huck Institutes of Life Sciences, The Pennsylvania State University, University Park, PA, United States of America; 6 Department of Animal Science, The Pennsylvania State University, University Park, PA, United States of America; Guru Angad Dev Veterinary and Animal Sciences University, INDIA

## Abstract

Zoonotic tuberculosis in humans is caused by infection with bacteria of the *Mycobacterium tuberculosis* complex acquired from animals, most commonly cattle. India has the highest burden of human tuberculosis in the world and any zoonotic risk posed by tuberculosis in bovines needs to be managed at the source of infection as a part of efforts to end human tuberculosis. Zoonotic tuberculosis in humans can be severe and is clinically indistinguishable from non-zoonotic tuberculosis. As a consequence, zoonotic tuberculosis remains under-recognised and the significance of its contribution to human tuberculosis is poorly understood. This study aimed to explore any association between bovine density, bovine ownership, and human tuberculosis reporting in India using self-reported tuberculosis data in households and officially reported tuberculosis cases while controlling for common confounders for human tuberculosis. We find an association between human tuberculosis reporting, bovine density and bovine ownership in India. Buffalo density was significantly associated with an increased risk of self-reported tuberculosis in households (odds ratio (OR) = 1.23 (95% credible interval (CI): 1.10–1.39) at household level; incidence rate ratio (IRR) = 1.17 (95% CI: 1.04–1.33) at district level), while cattle density (OR = 0.80, 95% CI: 0.71–0.89; IRR = 0.78, 95% CI: 0.70–0.87) and ownership of bovines in households (OR = 0.94, 95% CI: 0.9–0.99; IRR = 0.67, 95% CI: 0.57–0.79) had a protective association with tuberculosis reporting. It is unclear whether this relates to differences in tuberculosis transmission dynamics, or perhaps an association between bovines and other unexplored confounders for tuberculosis reporting in humans. Our study highlights a need for structured surveillance to estimate the prevalence of tuberculosis in cattle and buffaloes, characterisation of *Mycobacterium tuberculosis* complex species present in bovines and transmission analyses at the human-animal interface to better assess the burden and risk pathways of zoonotic tuberculosis in India.

## Introduction

Tuberculosis (TB) is an infectious disease caused by members of the *Mycobacterium tuberculosis* complex (MTBC). In humans, it is one of the top ten causes of death worldwide, with an estimated 10 million new cases of infection every year [1]. Well-documented risk factors for human TB include environmental and health-related factors such as smoking, indoor air pollution, coinfection with HIV, diabetes, undernutrition, and alcohol use disorders, as well as broader social determinants such as poverty [1–3].

Tuberculosis is also a major disease in cattle and the most likely source of zoonotic transmission of TB to humans [4]. The true burden of zoonotic TB remains unknown. World Health Organization estimates between 69,800 and 235,000 new human TB cases annually are of zoonotic origin caused by *Mycobacterium bovis* (*M*. *bovis*), the primary causative agent of TB in cattle, often referred to as bovine TB (bTB) [5]. However, in some areas, there is growing evidence for presence of other *Mycobacterium* species in cattle, in particular *M*. *tuberculosis* and *M*. *orygis* [6, 7], and the true burden of zoonotic TB may be higher. The presence of the same *Mycobacterium* species in both human and bovine populations could result from animals being a reservoir for human infection, humans acting as maintenance hosts with occasional spillover to bovines, or infection being endemic in both human and animal populations [8].

India has the largest population of bovines in the world with nearly 300 million cattle and buffaloes [9], but currently has no systematic surveillance or control strategy for bTB. A recent meta-analysis [10] suggests that the prevalence of bTB is variable, with an estimated pooled bTB prevalence of 7.3% (95% CI 5.6, 9.5) at the animal level in cattle and buffaloes. This estimate would translate nationally to approximately 21.8 million infected bovines, suggesting India may have the highest bTB burden globally in terms of number of infected animals [10]. As a chronic infection, and given the size of the bovine population in India, the relatively low prevalence may still pose a considerable risk for zoonotic transmission. Zoonotic TB transmission can occur through consumption of unpasteurised milk and milk products, direct contact with infected cattle or from processing contaminated carcases (reviewed in Grange [11]). Approximately 60–65% of milk produced in India is sold unpasteurised or consumed locally [12, 13] and Saidu et al. [13] found that only 39% of livestock owners regularly boiled raw milk before consumption. *Mycobacterium spp*. have been found in milk samples from cattle in India (Aswathanarayana et al. [14], as cited by Srinivasan et al. [10]) and, considering the high proportion of milk that is sold unpasteurised, pose a potential public health risk [10]. Moreover, the religious significance of cattle in India may lead to increased direct human-cattle contact [15], presenting opportunities for aerosol transmission of zoonotic TB [16, 17].

India has the highest burden of human TB in the world, accounting for 26% of estimated incident cases worldwide [1]. To achieve the World Health Organization aim of ending global TB, the zoonotic risk posed by bTB needs to be considered and addressed at the source of infection in the animal population [18]. The impact of zoonotic bTB is notoriously difficult to estimate [19]. In absence of systematic prevalence estimates of bovine and zoonotic TB in India, we set out to perform a preliminary exploratory risk factor analysis to characterise the association between bovine density, ownership of bovines and the incidence of TB in India as a guide to inform the design of future studies to assess the zoonotic risk of bTB.

## Methods

### Data sources

We use two sources of data for human tuberculosis incidence in India: officially notified district level data and self-reported household data. TB is a notifiable disease in India, with official notifications collated by the Government of India in the Nikshay database [20]. Notification data is available at the (administrative) district level. However, there is likely to be considerable geographic variation in the rates of reporting due to differences in practice in private and public clinics [21, 22]. The Demographic and Health Surveys (DHS) program routinely carry out household surveys in India including questions on tuberculosis [23]. In comparison to the official TB notification data, the DHS household dataset from the National Family Health Survey (NFHS-4) provides a sample of incidence nationally at a finer scale and may capture officially unreported cases from private healthcare settings, but at the cost of a less specific case definition. Given the limitations of both datasets and potential for bias, for this study we use three datasets derived from these two sources to examine risk factors at the household and district levels: 1. self-reported TB at the household level from the National Family Health Survey (NFHS-4) 2015–16 [23], 2. official TB notifications collated by the Government of India in the Nikshay database at a district level [24], and 3. self-reported TB in households from the National Family Health Survey (NFHS-4) 2015–16 [23] aggregated to the district level to allow a more direct comparison to the aggregated Nikshay notification data. Data sources of bovine measures and confounder variables considered in the analysis are described in Tables 1 and 2. Island territories Andaman and Nicobar islands as well as Lakshadweep were excluded from the analysis to allow for assessing spatial variation, which requires a continuous spatial field.

**Table 1:** Description and summary statistics of variables considered for the household level model of self-reported human tuberculosis in households in India. Data were sourced from a survey covering 601 509 households with ever-married women in India in 2015 [23].

Variable	Description	Variable summary	% of households	Source
TB present	self-reported TB in household	no = 586148	98.55	IIPS and ICF [23]
		yes = 8601	1.45	
total households	households interviewed with self-reported TB status	594749		IIPS and ICF [23]
*Exposure variables*				
bovines	bovines (cattle or buffaloes) owned by household	no = 371527	62	IIPS and ICF [23]
		yes = 223222	38	
cattle density	cattle density (number of cattle per km^2^) in district	min = 0.03, median = 57, max = 315		Ministry of Agriculture [25]; area^-1
buffalo density	buffalo density (number of buffalo per km^2^) in district	min = 0, median = 18, max = 454		Ministry of Agriculture [25]; area^-1
*Confounding variables*				
human density	human density (human population per km^2^) in district	min = 0.9, median = 367, max = 169906		human population/ district area
residence type	urban or rural residence	urban = 173576	29	IIPS and ICF [23]
		rural = 421173	71	
household size	number of household members	min = 1, median = 4, max = 41		IIPS and ICF [23]
IAP	exposure to indoor air pollution from cooking with solid fuels (agricultural crop, animal dung, wood, straw, shrubs, grass, coal, lignite, or charcoal) in the house	unexposed = 318082	53	IIPS and ICF [23]
		exposed = 276667	47	
			
smokes indoors	smoking indoors, where yes includes smoking indoors ’daily’, ’weekly, ’monthly’ and ’less than monthly’ and no corresponds to ’never’	no = 317641	53	IIPS and ICF [23]
		yes = 277108	47	
			
health scheme	a household member is covered by a health scheme or health insurance	no = 439349	74	IIPS and ICF [23]
		yes = 155400	26	
wealth index	index of wealth, where high is reformatted from ’richer’ or ’richest’ and low is ’poor’ or ’poorest’ in the NFHS-4 survey	high = 214157	36	IIPS and ICF [23]
		middle = 120501	20	
		low = 260091	44	

**Table 2:** Description and summary statistics of variables considered for the two district level models of human tuberculosis in India.

Variable	Description	Variable summary		Source
TB notifications	official human TB notifications by district	min = 1, median = 1960, max = 37951		Central TB Division [24]
human population	human population by district	min = 8004, median = 1510075, max = 24981566		Census Organization of India [44]
area	area of each district (km^2^)	min = 10, median = 3853, max = 47762		IIPS and ICF [23]
HH TB	households with self-reported TB in district	min = 0, median = 11, max = 69		TB present by district
households	households interviewed in district with self-reported TB status	min = 593, median = 881, max = 2443		total households by district
*Exposure variables*				
HH bovines	number of households with bovines	min = 0, median = 384, max = 906		bovines: yes by district
cattle density	cattle density (number of cattle per km^2^) in district	min = 0.03, median = 57, max = 315		Ministry of Agriculture [25]; area^-1
buffalo density	buffalo density (number of buffalo per km^2^) in district	min = 0, median = 18, max = 454		Ministry of Agriculture [25]; area^-1
*Confounding variables*				
human density	human density (human population per km^2^) in district	min = 0.9, median = 365, max = 169906		human population/district area
HH urban	number of urban households in district	min = 0, median = 175, max = 1686		residence type: urban by district
HH size	mean household size in district	min = 3.3, median = 4.7, max = 6.9		mean household size by district
HH IAP	number of households exposed to indoor air pollution	min = 1, median = 452, max = 1249		IAP: exposed by district
HH smoking	number of households with indoor smoking	min = 81, median = 400, max = 1584		smokes indoors: yes by district
HH health scheme	number of households in a health scheme	min = 5, median = 162, max = 1262		health scheme: yes by district
HH low wealth	number of households with low wealth index	min = 3, median = 426, max = 1101		wealth index: low by district
MPI	global Multidimensional Poverty Index by district	min = 0, median = 0.11, max = 0.40		OPHI [26]

### Model development

#### Response variables

An exploratory risk factor analysis was performed for the potential association between TB occurrence, bovine density and ownership in India by fitting three regression models at the household and district levels. At household level (model 1), the response variable was self-reported TB status in households, which was assessed through the question, *“Does any usual resident of your household suffer from tuberculosis*?*”* in the NFHS-4 survey [23]. An affirmative answer resulted in classification as a household with self-reported TB.

For the district level models, the response variable was either the number of official TB notifications (model 2) or self-reported households with TB aggregated at district level (model 3). Population size or surveyed number of households, respectively, were included as offset terms in order to model incidence rates rather than case counts. A negative binomial response was assumed to allow for over-dispersion.

#### Exposure variables

For each regression model, two models for bovines related measures and any association with tuberculosis reporting in humans were considered: bovine ownership in households and bovine density, where cattle density and buffalo density were considered separately. While household bovine ownership may be an indicator of contact with bovines in households, the measure will not include bovines kept in farms and gaushalas (where unproductive and retired cows are cared for). Bovine density captures larger herds in farms in addition to household owned bovines. Furthermore, the bovine density measures allow to distinguish between cattle and buffaloes, which was not possible based on the household data. Cattle and buffalo were considered individually as, in addition to being separate species, which could result in different disease transmission dynamics, they have distinct roles in the Indian society. Bovine ownership in households was extracted from the NFHS-4 household data and aggregated at a district level for the district level analysis. To obtain the density of bovines at district level, the number of cattle and buffaloes in districts as per the 19^th^ livestock census [25] was divided by the district area (Tables 1 and 2).

#### Confounding variables

Common risk factors associated with human TB were incorporated as confounding variables in the models (household level: Table 1; district level: Table 2). We started with a pre-selected set of confounder variables of the NFHS-4 household data identified by a literature review of possible risk factors for human TB (Table 1). To reduce the number of variables in the model and aid interpretation, several confounding variables were combined or discretised.

At the district level, these household level variables were aggregated to create new district level variables. In addition to variables measured within the NFHS-4 survey, we collated district level data on human density (Table 2). We also considered an aggregate measure of economic status, the Global Multidimensional Poverty Index (MPI) [26, 27] to attempt to adjust for this important risk factor for human disease. All continuous exposure and confounding variables at the district level were transformed to be on a comparable scale by using zero mean and divided by 2 standard deviations.

#### Variable correlation

The exposure and confounding variables were screened for collinearity with other variables (S1 Fig). There was evidence of multicollinearity between wealth index status and exposure to indoor air pollution (IAP) at both a household level (Cramer’s V = 0.52) and district level (Pearson correlation coefficient, *r* = 0.78). As a result, IAP was removed from both the household level and district level models. Furthermore, at district level, a strong positive correlation was found between the number of households with a low household wealth index and MPI (Pearson *r* = 0.84). Household wealth index and MPI also had very similar incidence rate ratios (IRR) for both response variables considered at district level. For consistency with the household level model, we therefore chose to drop MPI in favour of the aggregated household wealth index for the district level models.

### Data analysis

A univariable assessment was carried out on all proposed exposure and confounding variables to determine which risk factors were associated with TB reporting alone. All variables were considered biologically relevant and were retained in the multivariable regression analyses for consistency across the three regression models. A binomial logistic regression model was fitted for self-reported TB status at household level (model 1) and a negative binomial regression model for officially notified TB cases (model 2) and aggregated self-reported TB in households (model 3) at district level. For each model, one of the bovine exposure measures, bovine ownership (a) or cattle and buffalo density (b), were assessed together with the confounding variables listed in Table 1 at household level and Table 2 at district level. The six models considered with the three different response variables and two different bovine exposure measures are summarised in S1 Table.

The candidate multivariable models of self-reported household TB at district level demonstrated unexplained spatial variation with significant spatial clustering of the residual error. Due to the differences observed in the outputs of the three considered regression models, where the direction and significance of the association between the exposure variables and TB reporting varied depending on the data and spatial resolution used, we went on to explore whether this unexplained spatial variation impacted on our results. We adapted a Bayesian hierarchical framework, R-INLA [28, 29], allowing to take into account both spatially unstructured random effects and unmeasured spatial variation, accounting for the assumption that geographically close areas may be more similar than distant areas [30]. We specifically considered two forms of spatial random effect terms: independent identically distributed (IID) random effect accounting for unstructured variation in the data and spatial random effect using the Besag-York-Mollie (BYM) specification, which models both spatially structured residual effect as well as unstructured residual variation [30]. We used the marginal likelihood, deviance information criterion (DIC) and Watanabe-Akaike information criteria (WAIC) to select between the random effects and null baseline models.

All analyses were performed in R version 4.1.0 [31]. Maps for visualisation where produced in R [31–33] using shapefiles obtained from the Spatial Data Repository [34] and GADM database [35].

## Results

### Household level

Out of 594,749 households surveyed with self-reported TB status in the NFHS-4 data, 14.5 per thousand households reported at least one TB case within the household, 71% of households were located in a rural location, 47% of households reported smoking indoors, 38% had bovines, 26% were members of a health scheme, and 44% of households had a low wealth index. Variables considered and summary statistics are described in Table 1. All variables had *P*-values of <0.05 in the univariable analysis of self-reported TB in households (model 1), apart from human density in the district, which had a *P*-value of 0.12 but we choose to force into the model given that it is a useful design parameter for studies on zoonotic transmission.

### District level

**Fig 1:**
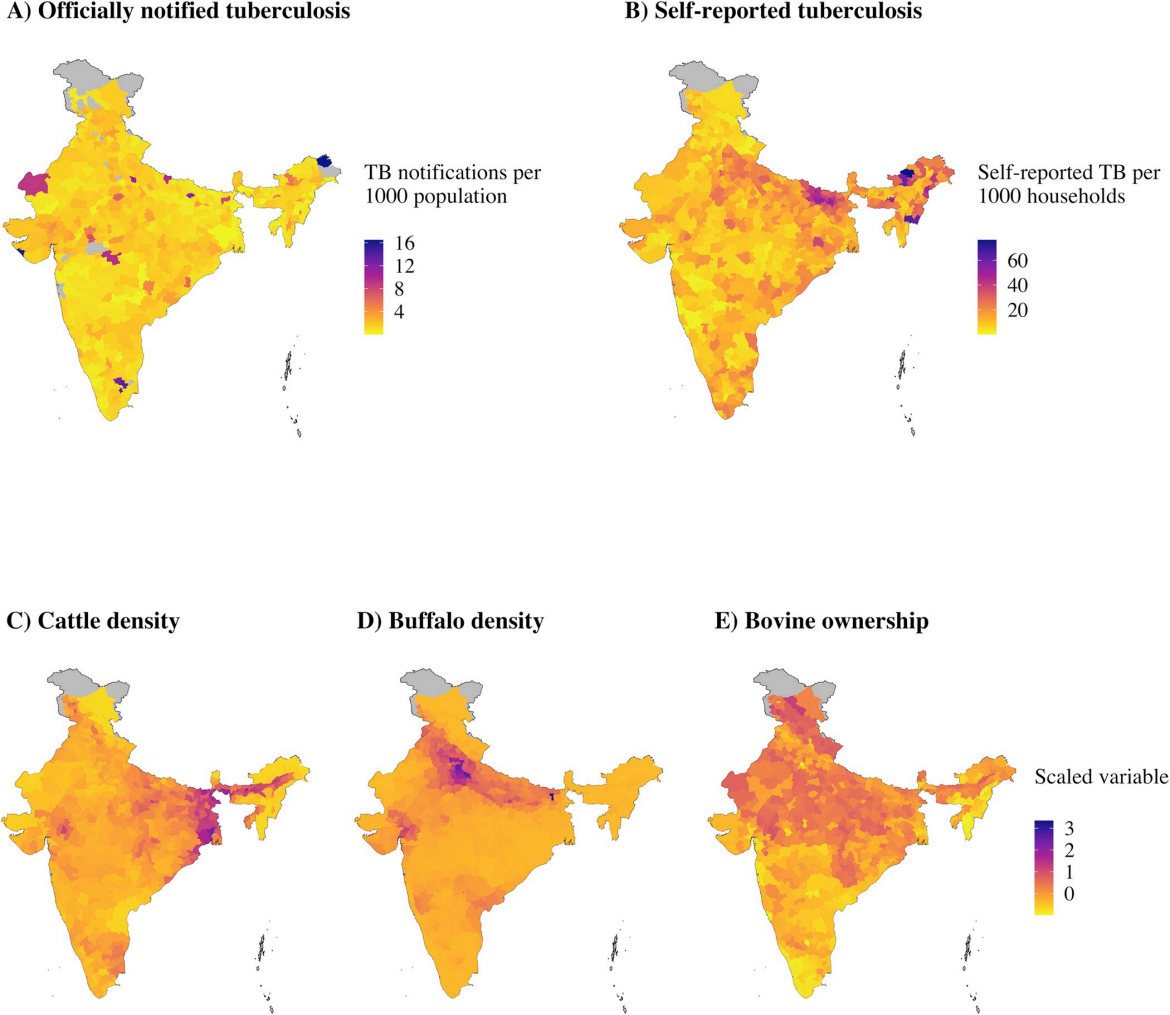
A) Officially notified tuberculosis cases per 1000 human populations and B) households with self-reported tuberculosis per 1000 households at district level in India and C) cattle density (animals per km^2^), D) buffalo density (animals per km^2^) and E) proportion of households with bovines, which have been standardised to the same scale for comparison. Districts with no data and excluded districts are indicated in grey.

**Fig 2:**
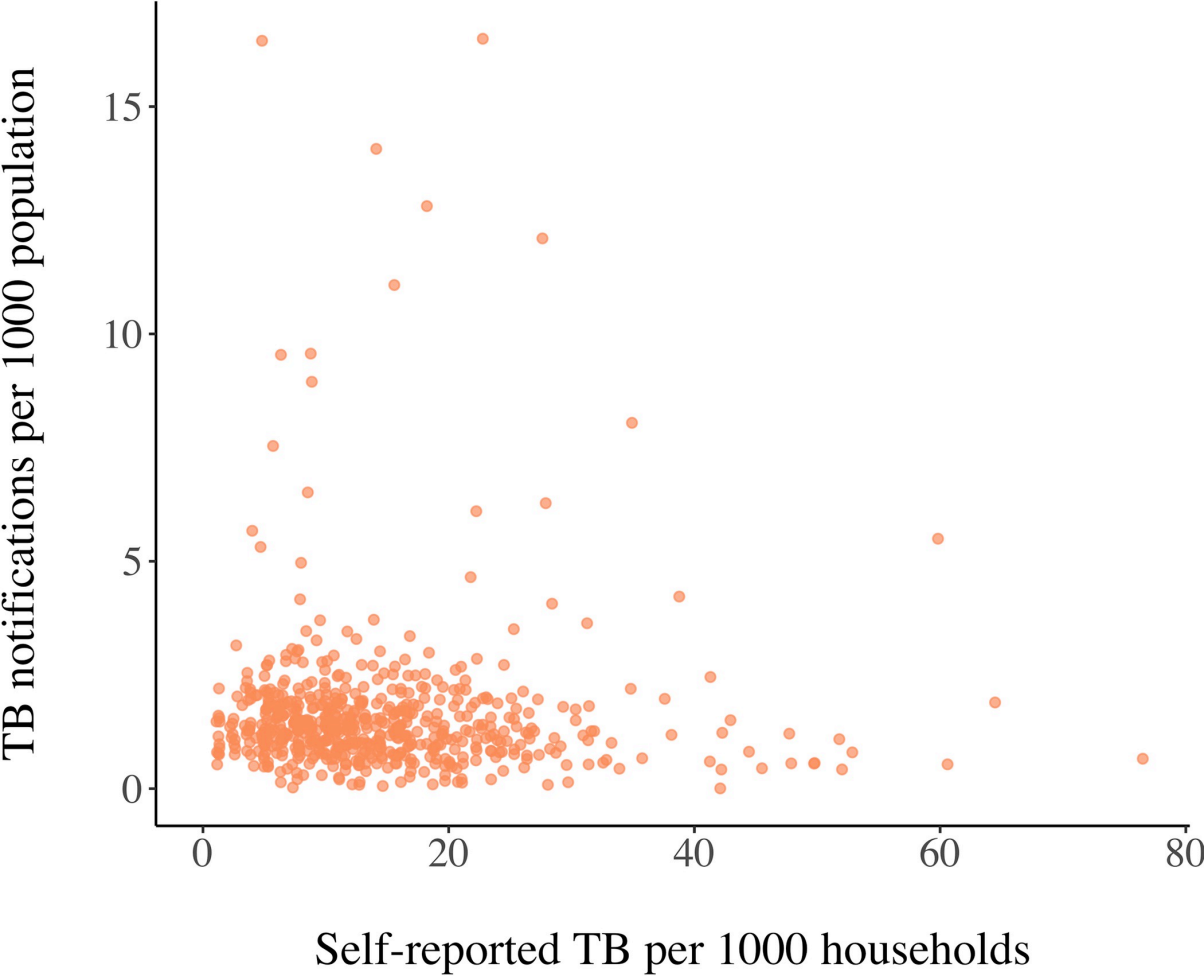
Visualisation of officially reported tuberculosis cases in individuals and households with self-reported tuberculosis at district level in India.

At district level, tuberculosis case notifications from the officially reported database ranged from 0.005–17 TB cases per thousand population, with no available TB notification data for 33 districts (Fig 1A). Self-reported TB in households aggregated at district level ranged from 0–77 per thousand households (Fig 1B). There did not appear to be any association between the number of officially reported TB cases in and households with self-reported TB in districts (Fig 2). The correlation between per household self-reports and per capita official TB notifications was negative (cor = -0.023) but indistinguishable from zero (*P*-value = 0.57, Pearson’s product moment correlation coefficient). Cattle and buffalo densities were concentrated in different parts of the country (Fig 1C and 1D), with bovine ownership in households being higher in Northern, North Eastern, Central and Eastern and India than Western and Southern India (Fig 1E).

Exposure and confounding variables considered in the analysis are summarised in Table 2. In the univariable analysis, all had a *P*-value of <0.05 in both district level models, apart from human density (IRR = 0.95, 95% CI: 0.89–1.01, *P*-value = 0.13) and number of households with bovines (IRR = 0.98, 95% CI: 0.94–1.02, *P*-value = 0.3) for self-reported TB in households at district level (model 3).

### Spatial models

Residual errors from the final multivariable models of self-reported household TB at district level were found to exhibit significant spatial clustering. To adjust for unexplained spatial variation, we used a Bayesian hierarchical approach, R-INLA [28, 29], to incorporate spatial random effects into our models. The BYM model, which takes into account both undescribed random variation and spatially structured variation, was favoured by all selection criteria for the three models and two different bovine exposure measures (S1 Table)

**Fig 3:**
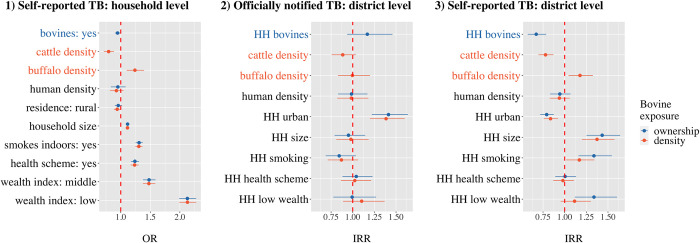
Mean fixed effects and 95% credible interval of potential risk factors for model 1) self-reported tuberculosis in households, model 2) officially notified tuberculosis at district level, and model 3) households with self-reported tuberculosis at district level in India. For each model, one out of two bovine exposure measures were assessed: bovine ownership in households (in blue) or cattle and buffalo density (in orange). Please note different axes for 1) and 2–3). OR = odds ratio, IRR = incidence rate ratio.

At a household level (model 1), buffalo density was associated with an increased risk of self-reported TB in households, while the probability of a household reporting TB significantly decreased with bovine ownership and cattle density (Table 3, Fig 3). Similarly, at a district level, buffalo density was associated with increased self-reported TB in households (model 3) and both household ownership of bovines and cattle density were protective against self-reported TB (Table 4, Fig 3). For officially notified TB cases, only the number of urban households in a district was significantly associated with an increased probability of TB reporting, while none of the other variables were statistically significant (Table 4, Fig 3).

**Table 3:** Posterior estimates of mean fixed effects, standard deviation (SD), odds ratio (OR) and 95% credible interval (CI) from Bayesian inference (INLA) of self-reported human tuberculosis in households (model 1) for the two exposure measures bovine ownership in households (A) or cattle and buffalo density (B) and confounding variables.

Variable	A) bovine ownership			B) bovine density		
	*Mean (SD)*	*OR*	*95% CI*	*Mean (SD)*	*OR*	*95% CI*
bovines: yes	-0.06 (0.03)	0.94	0.9–0.99	-	-	-
cattle density	-	-	-	-0.23 (0.06)	0.80	0.71–0.89
buffalo density	-	-	-	0.21 (0.06)	1.23	1.10–1.39
human density	-0.05 (0.07)	0.95	0.83–1.08	-0.08 (0.06)	0.92	0.82–1.05
residence type: rural	-0.04 (0.03)	0.96	0.90–1.02	-0.06 (0.03)	0.94	0.88–1.00
household size	0.11 (0.00)	1.12	1.11–1.12	0.11 (0.00)	1.12	1.10–1.12
smokes indoors: yes	0.27 (0.02)	1.31	1.25–1.37	0.27 (0.02)	1.31	1.25–1.37
health scheme: yes	0.21 (0.03)	1.23	1.17–1.31	0.21 (0.03)	1.23	1.17–1.30
wealth index: middle	0.39 (0.04)	1.48	1.38–1.59	0.39 (0.04)	1.48	1.37–1.58
wealth index: low	0.75 (0.03)	2.12	1.99–2.28	0.76 (0.03)	2.14	1.99–2.28

**Table 4:** Posterior estimates of mean fixed effects, standard deviation (SD), incidence rate ratio (IRR) and 95% credible interval (CI) from Bayesian inference (INLA) of officially notified tuberculosis cases in humans (model 2) and self-reported tuberculosis in households (model 3) at district level in India. For each model, one out of two bovine exposure measures were assessed: A) bovine ownership in households or B) cattle and buffalo density.

Variable	Model 2: officially notified TB cases						Model 3: self-reported TB in households at district level					
	A) bovine ownership			B) bovine density			A) bovine ownership			B) bovine density		
	*Mean (SD)*	*IRR*	*95% CI*	*Mean (SD)*	*IRR*	*95% CI*	*Mean (SD)*	*IRR*	*95% CI*	*Mean (SD)*	*IRR*	*95% CI*
HH bovines	0.16 (0.11)	1.17	0.94–1.46	-	-	-	-0.40 (0.08)	0.67	0.57–0.79	-	-	-
cattle density	-	-	-	-0.12 (0.08)	0.89	0.76–1.04	-	-	-	-0.25 (0.06)	0.78	0.70–0.87
buffalo density	-	-	-	0.00 (0.09)	1.00	0.83–1.20	-	-	-	0.16 (0.06)	1.17	1.04–1.33
human density	-0.01 (0.09)	0.99	0.83–1.17	-0.02 (0.09)	0.98	0.82–1.18	-0.06 (0.06)	0.94	0.83–1.07	-0.06 (0.06)	0.94	0.83–1.06
HH urban	0.35 (0.07)	1.42	1.22–1.64	0.33 (0.07)	1.39	1.20–1.60	-0.23 (0.05)	0.80	0.72–0.87	-0.18 (0.05)	0.84	0.76–0.92
HH size	-0.05 (0.09)	0.95	0.79–1.14	-0.02 (0.10)	0.98	0.81–1.18	0.36 (0.07)	1.43	1.25–1.64	0.32 (0.07)	1.38	1.20–1.57
HH smoking	-0.17 (0.1)	0.84	0.69–1.04	-0.14 (0.10)	0.87	0.71–1.06	0.29 (0.07)	1.34	1.16–1.54	0.16 (0.07)	1.17	1.02–1.34
HH health scheme	0.04 (0.08)	1.04	0.88–1.23	0.02 (0.09)	1.02	0.86–1.21	0.00 (0.06)	1.00	0.89–1.13	-0.02 (0.06)	0.98	0.87–1.11
HH low wealth	-0.01 (0.13)	0.99	0.78–1.27	0.10 (0.11)	1.11	0.89–1.37	0.29 (0.09)	1.34	1.12–1.60	0.11 (0.08)	1.12	0.95–1.30

**Fig 4:**
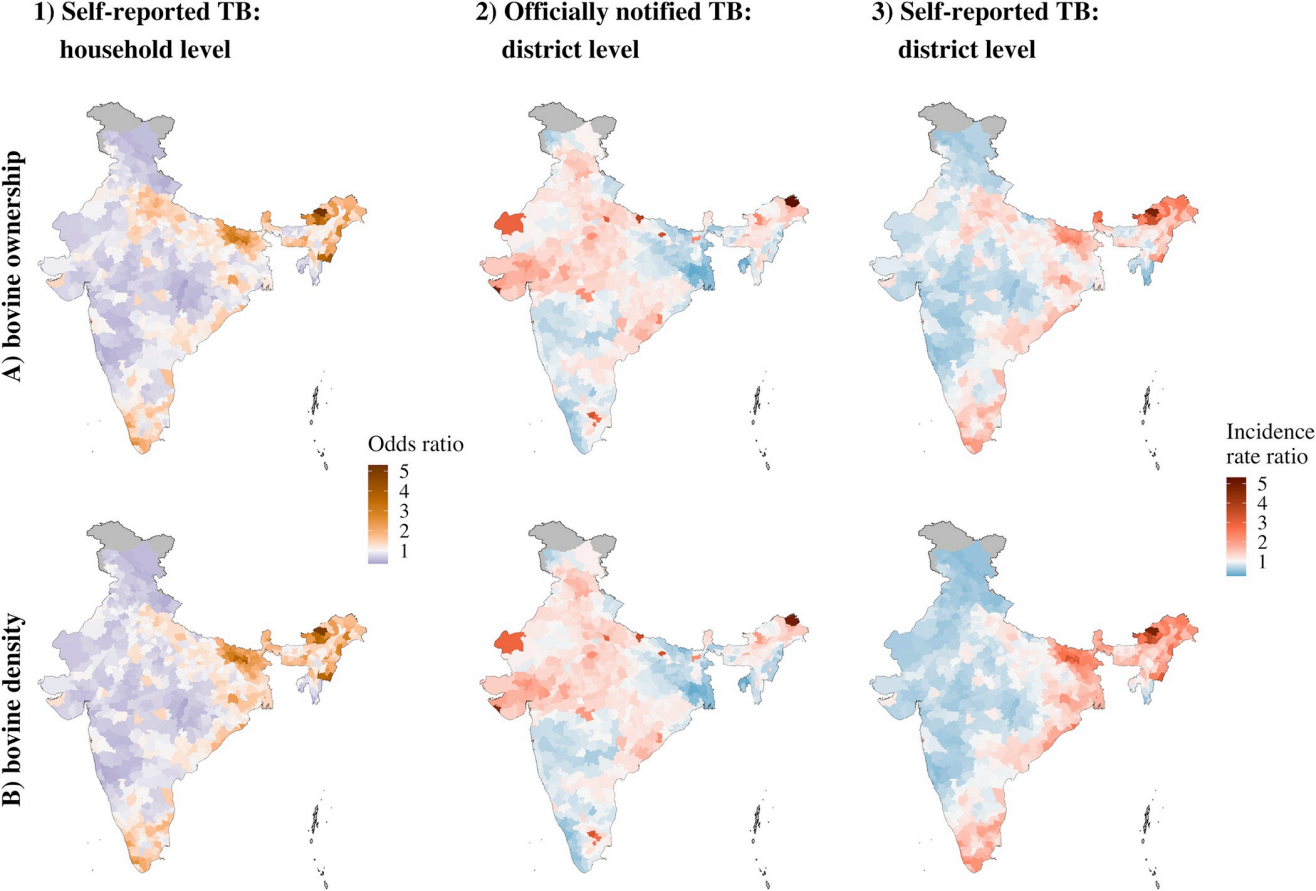
Estimated unexplained spatial variation in 1) self-reported tuberculosis in households, 2) officially notified tuberculosis at district level, and 3) self-reported tuberculosis in households at district level in India compared to the overall risk of tuberculosis reporting in the country after accounting for bovine ownership in households (A) or cattle and buffalo density (B) and cofounding variables. OR = odds ratio, IRR = incidence rate ratio.

The estimated unexplained spatial variation in TB reporting after accounting for the bovine exposure measures and the confounding variables was notably different between the models. Although all models estimated an increased probability of TB being reported in parts of North Eastern and Southern India, the IRR of the unexplained spatial effect was higher for officially reported TB in parts of Northern, Central and Western India while the same areas were associated with a decreased odds ratio (OR) and IRR for self-reported TB in households at household level and district level, respectively (Fig 4). The residual spatial variation between districts was wider for official TB notification (IRR range 0.2–5.3 and 0.3–5.0) than self-reported TB in households (IRR range 0.4–4.8 and 0.4–4.9), suggesting there was more unexplained spatial variation in the IRR of officially notified TB at district level after accounting for the described exposure and confounding variables.

## Discussion

In this exploratory study, we describe an association between human TB reporting, bovine density and bovine ownership in India. Buffalo density was significantly associated with an increased risk of self-reported TB in households both at a household and district level, while cattle density and ownership of bovines in households had a protective association with TB reporting. However, in the absence of bTB prevalence data in buffaloes and cattle, interpretation of the results is challenging. Since multiple confounders could affect TB occurrence, the effect size of bovine density and ownership on human TB should be interpreted cautiously. Although self-reported TB appears to be higher in areas with a higher buffalo density, bovine density and ownership may indirectly be measuring risk factors for human-to-human transmission.

Drivers of TB infection, diagnosis and reporting is context dependant. Although the confounding variables were selected based on previous research of risk factors for TB, there is uncertainty associated with the role of the selected variables in the local context and potential confounders which could affect the estimated risk associated with the assessed variables. Indeed, one of the limitations of the household level survey data is the inability to include any biometric risk factors, such as nutritional status, alcohol consumption, body mass index (BMI), or HIV status, which are commonly correlated with TB infection [3, 5, 36]. Furthermore, there may be relevant risk factors in this particular context that remain unknown and, therefore, cannot be accounted for. We have used spatial and random effect terms to adjust for unknown variation in TB reporting occurring between districts and across the country, respectively. However, unknown confounders affecting the exposure and confounding variables assessed are not considered and may, therefore, affect the estimated risk associated with covariates.

Associations between two variables could arise from a direct casual effect of one variable on the other or another confounding variable associated with both of them, either directly or indirectly [37]. An effect of bovine density or bovine ownership on tuberculosis reporting only suggests that there is an association between the exposure and response variables, but the intermediate steps through which bovines may influence tuberculosis reporting are not described in the model, and may be influenced by unknown risk factors associated with both tuberculosis reporting and bovine density or ownership. Consequently, bovines would appear to be associated with tuberculosis reporting when, in reality, tuberculosis reporting would be associated with an unknown risk factor which also influences or is influenced by bovines. For example, the lower rates of TB reporting associated with bovine ownership and cattle density may directly relate to TB transmission dynamics or may reflect an association between the exposure variables and another confounding protective factor, such as nutritional status.

As such, no conclusions can or should be made regarding whether variation in human TB reporting explained by buffalo density is due to buffalo-to-human TB transmission or increased human transmission due to confounding factors in areas with larger buffalo populations. In order to infer such conclusions, knowledge of the bTB infection status of the animals in the study is essential. However, the results of this assessment could be used to help identify study areas where zoonotic TB transmission may occur if present. For example, areas with higher buffalo density in Northern and north Central India (Fig 1D) could be targeted for case-control studies and testing of buffaloes for TB.

Questions assessing exposure to zoonotic risk factors would be a beneficial addition to epidemiological surveys. In the NFHS-4 [23], the survey question addressing bovine ownership was not included for the purpose of assessing zoonotic risk, but instead was part of a wider series of wealth questions exploring ownership of agricultural assets such as land and other livestock. More targeted questions assessing direct contact with bovines or consumption of unpasteurised milk would aid understanding of transmission dynamics and may help to distinguish the effect of bovine ownership from other confounders, which was a limitation here.

The differences observed in the unexplained spatial variation between officially reported TB and self-reported TB (Fig 4) after accounting for the bovine exposure measures and the confounding variables raises questions about structural differences in the reporting and representativeness of the data. There was no association between officially notified TB cases in individuals and self-reported TB cases in households at district level (Fig 2). Official notifications of a condition depend on cases accessing health care, diagnosis being made and confirmed diagnosis being reported. As such, suspected cases that receive treatment without a confirmed diagnosis are not reported. Indeed, India is estimated to have the highest gap between notified TB cases and estimated incidence of TB in the world [1]. Official notification of TB cases has been mandatory in India since 2012 [38]. However, notification rates, particularly within the private health sector remain low [39–41]. Furthermore, geographical distribution of official notifications can be biased by discrepancies in accessibility of healthcare facilities between states, leading to increased notifications in certain urban areas, such as Delhi, reflecting diagnoses in patients travelling from other areas [42]. The self-reported TB data in households provides an alternative estimate of the TB burden in India with a nationally representative sampling approach [21]. Nevertheless, household level assessment can also lead to missed events and for conditions that are commonly underreported, the risk of underreporting might be higher at household level than individual level [43]. Because of underdiagnosis of cases as well as stigma connected to TB, underreporting by respondents of the self-reported TB household survey is a concern and the true TB prevalence is likely to be higher [21].

So, to answer the question ‘Is bovine density and ownership associated with human tuberculosis in India?’: Yes, there appears to be an association between TB reporting and bovine ownership, cattle density or buffalo density in India. The direction and significance of the associations depend on the dataset and model used, but the presence of an association between bovines and TB reporting appears to remain even after adjusting for known TB risk factors and spatial random effects. However, the pathway of the association between bovines and tuberculosis reporting is unknown and, as a result, the role of bovines in TB occurrence is uncertain. Due to the high burden of tuberculosis in India as well as the socioeconomical and cultural importance of bovines in the country, the potential role of bovines in zoonotic TB transmission warrants further investigation. Robust surveillance to assess the prevalence of TB in cattle and buffaloes, characterisations of MTBC species present in bovine populations as well as carefully designed case-control studies in selected subpopulations to quantify the risk of TB transmission between humans and bovines are urgently needed to understand the burden of zoonotic TB in India.

## Supporting information

S1 Fig Variable correlation. A) Cramer’s V correlation between household level variables. B) Pearson correlation between district level variables. Variables are described in Tables 1 and 2.
(PNG)

S1 Table Model comparison. (PDF)
