# Supplementary figures and images for: Is bovine density and ownership associated with human tuberculosis in India?
Source: PLoS One. 2023 Mar 22;18(3):e0283357. doi: 10.1371/journal.pone.0283357 (PMC10032477; doi:10.1371/journal.pone.0283357)

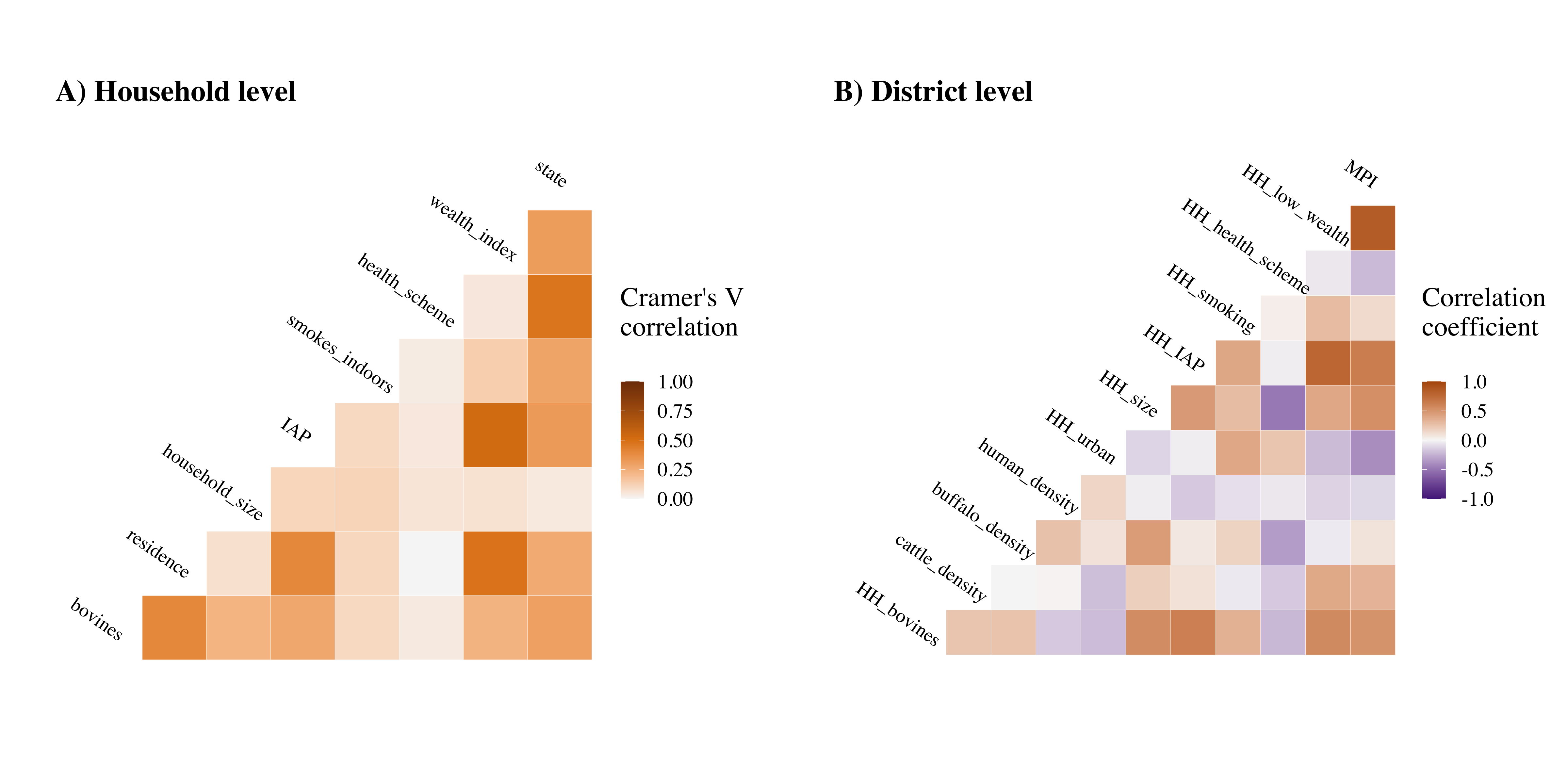

Supplement: S1 Fig — A) Cramer’s V correlation between household level variables. B) Pearson correlation between district level variables. Variables are described in Tables 1 and 2. (PNG) [file pone.0283357.s001.png]
