# Supplementary material for: Is bovine density and ownership associated with human tuberculosis in India?
Source: PLoS One. 2023 Mar 22;18(3):e0283357. doi: 10.1371/journal.pone.0283357 (PMC10032477; doi:10.1371/journal.pone.0283357)
Supplement: S1 Table — (PDF) [file pone.0283357.s002.pdf]

Supplementary material for

Is bovine density and ownership associated with human tuberculosis in India?

Authors

Katriina Willgert[1¶], Susie da Silva[1¶], Ruoran Li[2], Premanshu Dandapat[3], Maroudam Veerasami[4], Hindol Maity[4], Mohan Papanna[5], Sreenidhi Srinivasan[5], James L. N. Wood[1], Vivek Kapur[5, 6], Andrew J. K. Conlan[1]

[1]Disease Dynamics Unit (DDU), Department of Veterinary Medicine, University of Cambridge, United Kingdom;
[2]Department of Epidemiology, Harvard T.H. Chan School of Public Health, Boston, MA, United States
[3]ICAR-Indian Veterinary Research Institute, Eastern Regional Station, Kolkata, West Bengal, India
[4]CisGen Biotech Discoveries Pvt Ltd, Chennai, India
[5]Huck Institutes of Life Sciences, The Pennsylvania State University, University Park, PA, United States
[6]Department of Animal Science, The Pennsylvania State University, University Park, PA, United States

Supplementary material: Tables

Table S1. Overview and comparison of the models considered. Three response variables were assessed: 1. self-reported TB in households, 2. officially notified TB cases, and 3. self-reported TB in households aggregated at district level. For each model, two bovine exposure measures were explored: bovine ownership in households or cattle and buffalo density in districts. Models with and without spatial random effects were compared, where the Besag-York-Mollie (BYM) specification considers both spatially structured as well as unstructured residual variation, the independent identically distributed (IID) random effect accounts for unstructured variation, and the null model includes no random effect.

Model	Exposure	Spatial resolution	Family	Spatial effect	MLIK	DIC	WAIC
Model 1a: self-reported TB in households	Bovine ownership	Household	Binomial	BYM	-43118	86107	86095
				IID	-43363	86210	86181
				null	-44027	87954	87953
Model 1b: self-reported TB in households	Bovine density	Household	Binomial	BYM	-43115	86103	86090
				IID	-43364	86206	86177
				null	-44035	87956	87955
Model 2a: officially notified TB cases	Bovine ownership	District	Negative binomial	BYM	-4988	10017	10014
				IID	-5141	10116	10113
				null	-5169	10252	10263
Model 2b: officially notified TB cases	Bovine density	District	Negative binomial	BYM	-4994	10022	10021
				IID	-5146	10120	10119
				null	-5171	10246	10257
Model 3a: self-reported TB in households at district level	Bovine ownership	District	Negative binomial	BYM	-1886	3765	3756
				IID	-2103	4118	4121
				null	-2103	4119	4121
Model 3b: self-reported TB in households at district level	Bovine density	District	Negative binomial	BYM	-1894	3783	3784
				IID	-2144	4036	4087
				null	-2144	4189	4192

MLIK = marginal likelihood, DIC = deviance information criterion, WAIC = Watanabe-Akaike information criteria (WAIC)
